# Supplementary material for: Novel height estimation formula that accounts for the effects of aging based on lumbar length measurements in postmortem CT images
Source: PLoS One. 2026 Feb 3;21(2):e0339125. doi: 10.1371/journal.pone.0339125 (PMC12867242; doi:10.1371/journal.pone.0339125)
Supplement: S1 Table — (DOCX) [file pone.0339125.s003.docx]

Supplemental Table 1. Results of correlation analysis between residuals derived from each height estimation formula and age.

|  | R | p-value |
| --- | --- | --- |
| ALV1 | -0.375 | 0.001 |
| CLV1 | -0.307 | 0.006 |
| PLV1 | -0.292 | 0.009 |
| ALV2 | -0.231 | 0.042 |
| CLV2 | -0.161 | 0.158 |
| PLV2 | -0.134 | 0.242 |
| ALV3 | -0.308 | 0.006 |
| CLV3 | -0.280 | 0.013 |
| PLV3 | -0.212 | 0.062 |
| ALV4 | -0.219 | 0.054 |
| CLV4 | -0.251 | 0.027 |
| PLV4 | -0.254 | 0.025 |
| ALV5 | -0.323 | 0.004 |
| CLV5 | -0.249 | 0.028 |
| PLV5 | -0.252 | 0.026 |
| SALV | -0.214 | 0.060 |
| SCLV | -0.176 | 0.123 |
| SPLV | -0.142 | 0.214 |
| LVTL | -0.014 | 0.906 |
| SB | -0.474 | <0.001 |

R, Spearman's rank correlation coefficient; ALV, anterior margin height of lumber vertebra; CLV, central height of lumber vertebra; PLV, posterior margin height of lumber vertebra; SALV, the sum of ALV; SCLV, the sum of CLV; SPLV, the sum of PLV; LVTL, the lumbar vertebra total length; SB, sternal bone. The specific lumbar vertebrate number is indicated in each dataset measurement.
