# Supplementary material for: Novel height estimation formula that accounts for the effects of aging based on lumbar length measurements in postmortem CT images
Source: PLoS One. 2026 Feb 3;21(2):e0339125. doi: 10.1371/journal.pone.0339125 (PMC12867242; doi:10.1371/journal.pone.0339125)
Supplement: Attachment — Submitted filename: Response to Reviewers PLoS One.doc [file pone.0339125.s009.doc]

Dear Editor and Reviewers,

We thank you for your critical and instructive comments. We have read the comments carefully, revised the manuscript in response, and highlighted the modified points.

**Answers to Reviewer #1:**

*1. 74-76 & Supplemental Figure 1 - this seems a little awkward and should probably be moved to the results or completely removed from the manuscript, since it's not really the focus of the study. I see that it was added as a response to a reviewer, but I really don't think it's necessary to include more variables. If you do decide to keep this, make sure you also add the appropriate information to the Materials and Methods section (i.e., the information about how the measurements were taken, etc), and the results should be added to the tables (but I really think it's best just to remove it).*

As you suggested, the relevant text and Supplemental Figure 1 have been removed from the revised manuscript (see **second paragraph of Introduction**).

*2.* *Table 1 - it's not clear why Age is presented twice, and why the bottom three rows only have 244 individuals. I'm guessing that this is because the last three rows represent the sample associated with the sternal measurements, which is different from the sample used for lumbar measurements. If so, this should be clearer in the table/paper.*

As you pointed out, the sample sizes used for lumbar spine length measurement and sternum length measurement differ. Therefore, to clarify the text for the readers, the revised version separates the list of samples used for lumbar spine length measurement into Table 1 and the list of samples used for sternum length measurement into Table 2. Additionally, the reason for the differing sample sizes has been added to the text (see **Tables 1 and 2,** **pages 5-6, lines 118-120 of Materials**).

*3. l.235-236 & 237-238 - the p-value is not a direct probability of significance, but rather the probability of observing the data given the null hypothesis is true.*

We have deleted the relevant passage and revised it with a different expression (see **pages 19-20, lines 338-346 of Results**).

*4. Table 4 - I'm more interested in the actual estimates (coefficients) of the regression model than the p-values since these are more indicative of the effect of age, so these should be presented as well. The table should also present the multiple R-squared value, not the correlation coefficient (R).*

As you suggested, we included the R-squared value instead of the R value in Table 4 (see **Table 4**).

*5. Model evaluation: Given that this study is assessing the prediction ability of the model, it would be better to highlight the standard error, which is more indicative of prediction accuracy than the r-squared. Even better model evaluation would be to separate the data into training and prediction sets, and/or to use some form of cross-validation. Without this kind of evaluation it's not really possible to talk about prediction accuracy. It's also important to address the assumptions of linear regression. Are the residuals roughly normally distributed, is there independence of observations, do the residuals have constant variance (homoscedasticity), and is there a linear relationship between the predictor and response variables?*

As suggested, we emphasized not only a high R² value but also a low SEE to evaluate the predictive capability of the model we created (see **page 8, lines 179-181 of Statistical Analysis, pages 13-14, lines 250-258 of Height estimation formula, page 22, lines 369-375 of Discussion**). Additionally, to ensure proper model evaluation, we split the dataset into training and validation sets to assess predictive accuracy (see **pages 5-6, lines 102-127 of Materials, page 8, lines 183-185 of Statistical analysis, Figure 1, Tables 1 and 2, page 18, lines 307-312 of Validation test, page 22, lines 372-375 of Discussion**). Further, to address the assumptions of linear regression (see **page 8, lines 174-179 of Statistical analysis, page 14, lines 258-261 of Height estimation formula)**, we analyzed whether the residuals (1) are normally distributed (see **Table 5**), (2) are independent (see **Table 5**), (3) have constant variance (homoscedasticity) (see **Figure 5**), and (4) exhibit linearity between standardized residuals and standardized predicted values. (see **Figure 6**). To perform statistical analyses more efficiently, the statistical software was changed from Excel Statistics to R (see **pages 7, lines 163-164 of Statistical analysis**).

*6. Data availability - There is a discrepancy between the data availability statement in the manuscript and the statement in the metadata. The former states that data are available upon request (not sufficient according to PLOS Data Policy), while the latter states the data are fully available without restriction, and are available in the manuscript and supporting information (which seems to be the case). It would in any case be better to upload the data to a trusted data repository (like Zenodo or an institutional repository) to adhere to the FAIR principles. There also needs to be more documentation associated with the data file, like a README file or a data dictionary. The data in the file should also be more rectangular to facilitate re-use. There are currently many levels of headers, such as 'Lumbar length' > 'AVL1' > 'Measured value'. Such information about the data should be presented in a separate file (like the aforementioned README). Also, instead of 'Measured value', consider using the name of the actual measurement, e.g., ALV1.*

As suggested, we have uploaded the sample data used in this study to a trusted data repository (https://zenodo.org/records/17430849). We organized the data as suggested and also uploaded a README file (see **page 27, lines 481-482 of Data availability**).

*7. I tried to reproduce the analysis using the data provided in the supplementary materials (*minimal data set.xlsx*) and the methods described in the Methods section. I was able to match the summary statistics in Table 1, as well as the correlation coefficients and linear models for the sternal measurements (SB). However, none of the correlation coefficients or linear models of the lumbar measurements matched the presented results (Tables 2, 3, and 4). And it was quite a large difference, to the point where it would completely change the interpretation. Based on the results I got, the stature estimation method you propose will not work as there is little-to-no correlation between any of the variables and the height. Is it possible that the lumbar measurements in the data file are wrong (maybe they got mixed up before they were submitted)?*

We sincerely apologize. The data uploaded previously contain errors. We have confirmed that all analyses are reproducible before revising the submission and have uploaded the correct data*.*

**Other modifications:**

As you can see in the newly added Figure 3 in this revised version, the height distributions for men and women are clearly different. Therefore, whereas previous versions created separate height estimation equations for men and women from the outset, this revised version incorporates gender as an explanatory variable to create a single height estimation equation using combined data for both sexes (see **pages 7-8, lines 166-172 and Figure 3**). Additionally, following the switch to R for statistical analysis software, Figures 1, 3–7 were recreated using R instead of Excel software (see **page 7, line 163-164 and Figures 1, 3-7**).

We thank the reviewers for their comments and feel that the revised manuscript is much improved. We hope that you find the manuscript suitable for publication in *PLoS One*. We thank you in advance for your kindness and look forward to your favorable reply.

With my best regards,
