# Supplementary material for: Novel height estimation formula that accounts for the effects of aging based on lumbar length measurements in postmortem CT images
Source: PLoS One. 2026 Feb 3;21(2):e0339125. doi: 10.1371/journal.pone.0339125 (PMC12867242; doi:10.1371/journal.pone.0339125)
Supplement: Attachment — Submitted filename: R2 Response to Reviewers PLoS One.doc [file pone.0339125.s010.doc]

Dear Editor and Reviewers,

We thank you for your critical and instructive comments. We have read the comments carefully and revised the manuscript in response. In the revised manuscripts, we have highlighted the modified points.

**Comments to Reviewer #1:**

Thank you for your comments on our manuscript. We appreciate your understanding of the advantages and importance of our research.

**Answers to Reviewer #1:**

*1. Limited generalization: the study population is drawn from a specific geographic location (the author’s institute). This raises questions about the generalizability of the findings to other populations with different body proportions and age-related changes in stature. Further validation in diverse populations is needed.*

As you point out, the study is based on a limited sample of CT images of deceased cases in our prefecture. It is clear that there are ethnic differences in height, and, therefore, we have mentioned this point in the Limitations section that similar studies using samples from other ethnic groups are needed to generalize the present height estimation formula in the future. (please see **page 18, lines 336-339 of Limitations and new references [33]**).

*2.* *Sample: the study relies on postmortem CT images, while this provides a large sample size, it is important to consider potential differences between living and deceases individuals. Factors like postmortem changes in tissue and posture could introduce some bias into the measurement*

This study used postmortem CT images as samples, and the correct height was measured at the time of autopsy from the top of the head to the bottom of the feet in the supine position on the autopsy table, and is different from the method used to measure height in living persons, in addition to the influence of postmortem changes in tissue. Therefore, the height estimation formula developed in this study cannot be applied directly to a living person. Accordingly, we have added a mention about this in the Limitations section. (please see **pages 18, lines 340-345 of Limitations**).

*3. Lack of focus on Lumbar Spine: while the study focus on the lumbar spine is advantageous in some contexts, it also presents a limitation. Accurate measurement of the lumbar vertebrae requires CT imaging, which may not always be readily available. This limits the applicability of the method compared to simpler techniques using easily measurable skeletal elements.*

As you pointed out, postmortem CT images of the lumbar vertebrae were used to estimate height based on the equations obtained in this study, and CT images are not always readily available. This limits the practical application of this method compared to other methods using easily measurable bone, such as the humerus, radius, or ulna. Accordingly, we have discussed this in the Limitations section. (please see **page 18, lines 346-349 of Limitations)**.

*4. Measurement: Measuring vertebral heights and lengths from CT images can be subject to measurement error, particularly if image quality is suboptimal or if there are anatomical variations or pathologies present. The study does not explicitly address to potential impact of measurement error on the accuracy of the height.*

In accordance with your suggestion, the measurement of lumbar vertebrae length on CT images can potentially lead to measurement errors depending on the anatomical variations or pathologies/deformities of the corpse, which may affect the estimation of height. Accordingly, we have mentioned this in the Limitations section. (please see **pages 18-19, lines 350-353 of Limitations**).

**Comments to Reviewer #2:**

Thank you for your comments on our manuscript. We appreciate your understanding of the advantages and importance of our research.

**Answers to Reviewer #2:**

*1. It would be helpful to clarify the time interval between death and CT imaging, as postmortem decomposition can affect spinal measurements and height estimation.*

As you suggested, we added to the time interval between death and CT imaging in the Materials and Methods section. The average time interval was 4.8 days (range, 12 hours to 60 days; about 90% of cases within 7 days). Cases in which post-mortem decomposition clearly affected the reading were excluded from the sample. (please see **pages 5, lines 112-114 of Materials**).

*2. The study should specify whether postmortem CT is routinely used or only in specific cases. This will help assess the method's applicability in standard forensic investigations.*

In response to your suggestion, we have added text describing that, at our department, postmortem CT imaging are routinely performed in all autopsy cases except for completely skeletonized cadavers, and includes 200-250 cases per year. (please see **page 5, lines 101-103 of Materials**).

*3. Including more representative CT images would strengthen the methodology section, showcasing how measurements were taken across different age groups and cadavers.*

We agree with your suggestion and have added to Figure 2 a case with osteophyte formation and a case with old lumbar compression fracture in the lumbar vertebral body, which is difficult to measure. (please see **new Figure 2 and its caption in page 6, lines 129-138 of Measurement of lumber spine length of Results**).

*4. The study focuses only on the lumbar spine, but long bones like the femur and tibia also contribute to height. Including these measurements might improve the accuracy, especially in fragmented bodies. Incorporating measurements from long bones like the femur and tibia could enhance the reliability of height estimation, especially when the spine is fragmented or poorly preserved.*

Thank you for your comment. Due to the specifications of the CT equipment at our institution, the imaging range was from the head to the pelvic region, and it was not possible to measure lower limb bones such as the femur and tibia. On the other hand, as a preliminary study for this study, we attempted to create a height estimation formula based on the maximum length of the radius and ulna, which have traditionally been measured for height estimation in forensic practice, and we confirmed that in both cases, the estimated height tended to be estimated greater than actual height in elderly people. Accordingly, we have added text in the Introduction section and a scatterplot showing the difference between height estimates based on each bone length and the actual height as Supplemental Figure 1. (please see **page 4, lines 74-76 of Introduction, Supplemental Figure 1 and its caption in page 4, lines 89-93**).

*5. It’s important to mention whether radiographs were also performed. Since X-rays are more commonly used in forensic practice, it would be helpful to know if the formula can be applied to radiographs.*

In the present study, a sagittal section image of the lumbar vertebrae was created based on the 3D constructed CT image, and a height estimation equation was created from the values obtained by measuring the height of each lumbar vertebrae. This could also be applied using simple X-ray images (sagittal section) of the lumbar spine. As radiography has been common in the forensic practice for a long time, it is likely that radiography equipment is available even without CT equipment. Therefore, this estimation formula, which is thought to be able to estimate height even with simple X-ray images, can be applied widely in forensic practice. Accordingly, we have added this to the Discussion. (please see **page 17, lines 322-328 of Discussion**).

*6. The formula could be validated with data from living populations or a combination of living and postmortem data to ensure its generalizability across both groups.*

This study used postmortem CT images as samples, and the correct height was measured at the time of autopsy from the top of the head to the bottom of the feet in the supine position on the autopsy table. This method is different from that used to measure height in living persons, and accounts for the influence of postmortem changes in tissue. Therefore, the height estimation formula developed in this study cannot be applied directly to a living person. Accordingly, we have mentioned this in the Limitations section. (please see **pages 18, lines 340-345 of Limitations**).

*7. The impact of pathological conditions (e.g., fractures, scoliosis, osteoarthritis) on spinal height should be considered. Either excluding individuals with these conditions or accounting for them could make the findings more robust.*

Thank you for pointing this out. We focused on age-related changes in lumbar spine length, such as old lumbar vertebral compression fractures and lumbar scoliosis, and conducted this study in the belief that measuring lumbar spine length may provide an accurate height estimate that takes age-related changes into account. The original submission did not include the frequencies of old compression fractures and scoliosis in the sample, and, therefore, we have added these variables in the revised version (please see **page 5, lines 108-112 of Materials**). Moreover, we have added a new note in the Discussion stating that adding those cases to the sample has allowed us to develop an accurate height estimation equation that takes into account age-related height shortening. (please see **page 16, lines 300-304 of Discussion**).

*8. Further analysis of gender differences in lumbar spine measurements would help refine the height estimation formula for both males and females.*

Thank you for your comment. We initially created the height estimation equation separately for males and females. As a result, we found that for most of the measured lengths in this study, the correlation with height is greater when male and female data are combined than when these data are separated. However, we also found that for AVL1, AVL2, AVL3, and SAVL, the correlation with height was greater when male and female data were separated than when they were combined. We have added text to the Discussion to address this point. (please see **page 17, lines 314-319 of Discussion**).

**Other modifications:**

We extended the period during which we had access to the CT images due to the need to review the post-mortem CT images again in order to respond to the reviewers' comments (please see **page 5, lines 101-102**). Accordingly, we also applied to our institutional ethics committee to extend the duration of the study, and, as the extension was approved, we have attached the ethics review approval forms (original and translated versions) for the study extension. (please see **page 7, line 158** **and attached files named by “original ethics approval letter 2” and “approval document 2 (Translation)”**).

We thank the reviewers for their comments and feel that the revised manuscript is much improved. We hope that you find the manuscript suitable for publication in *PLos One*. We thank you in advance for your kindness and look forward to your favorable reply.

With my best regards,
