# Supplementary material for: Novel height estimation formula that accounts for the effects of aging based on lumbar length measurements in postmortem CT images
Source: PLoS One. 2026 Feb 3;21(2):e0339125. doi: 10.1371/journal.pone.0339125 (PMC12867242; doi:10.1371/journal.pone.0339125)
Supplement: Attachment — Submitted filename: R3 Response to Reviewers PLoS One.doc [file pone.0339125.s011.doc]

Dear Editor and Reviewers,

We thank you for your critical and instructive comments. We have read the comments carefully, revised the manuscript in response, and highlighted the modified points.

**Answers to Reviewer #3:**

*1. Data - great that they are now available on Zenodo, and in a form that is much easier to work with. I would recommend having all the raw data together in a single sheet in addition to the sheets containing the separated training and validation subsets.*

We are glad that you were able to successfully use the data we uploaded to Zenodo repository. As you pointed out, we uploaded the training subset and validation subset as separate files, each on a single sheet. Additionally, within the Folder containing the uploaded the R script, we have also included Text files created from the same Excel files (see Zenodo repository: **https://doi.org/10.5281/zenodo.17677851**).

*2.* *Analysis - since the revised analysis was conducted in R, it would be great if the R script could also be shared on the Zenodo repository. Note: if you create a new version of the Zenodo repository, remember to update the link (DOI) in the manuscript.*

We have uploaded all R scripts used for statistical analysis to the above Zenodo repository (a new version of the Zenodo repository). The files containing the R scripts were compressed into Folder (ZIP format) for upload. To reproduce the analyses, please download the entire Folder (named CT_project) and extract its contents. When using R, please select this Folder as your Working Directory in R Studio. Please use the “main_script” located in the folder named “scripts” within the same Folder (see Zenodo repository: **https://doi.org/10.5281/zenodo.17677851**).

*3. Results - Still some discrepancies in the correlation coefficients, but within a 5% margin which is reasonable.*

As you pointed out, after redoing all statistical analyses, we found minor errors in some Tables and have corrected them (see **Tables 3, 6 and Supplemental Table 1**).

*4. l. 341 & l. 345 - It seems a little arbitrary to state that -0.292 is a very weak negative correlation while -0.474 is a significant negative correlation. Unless you have another rule-of-thumb you can refer to, the former can probably be described as a small correlation and the latter as a medium correlation (according to Jacob Cohen's recommendation, 1992).*

As you pointed out, our interpretation of the strength of the correlation was somewhat arbitrary. Therefore, following the advice in the reference you suggested, we have revised the description (see **page 20, lines 339-343 of Results**). We have also added that reference to Reference lists as [32] (see **new** **Reference [32]**).

*5. Table 7 - This table is a bit confusing. The caption says regression analysis but the table presents a correlation coefficient. I'm guessing the caption should state 'Results of correlation analysis...' While there are no strong correlations between the measurements and Age, it may be worth noting that they are all negatively correlated (as would be expected from degenerative changes related to age), so the age estimation equation is consistently overestimating height as age increases, but to a lesser extent than sternal length. This table can probably also be moved to the supplementary materials.*

As you pointed out, the caption for Table 7 was incorrect since it represents a correlation analysis rather than a regression analysis. I have corrected it accordingly (see **the caption of Supplemental Table 1**). Furthermore, the explanation of the results in Table 7 was confusing, so we have revised them to appropriate descriptions (see **pages 19-20, lines 336-343 of Results**). Additionally, following your advice, we have moved Table 7 to Supplemental Table 1. (see **Supplemental Table 1**).

*5. R should be added to the references: R Core Team (2025). R: A Language and Environment for Statistical Computing. R Foundation for Statistical Computing, Vienna, Austria. https://www.R-project.org/.*

As you advised, we have added references on analysis using R (see **page 7, lines 164 of Statistical analysis and new Reference [29]**).

**Other modifications:**

In the residual analysis of regression analysis, we performed the Durbin-Watson test for testing independence in the manuscript we submitted last time. However, we found that this test method was incorrect because our research sample is not time-series data. We assessed independence (presence or absence of autocorrelation) by examining the scatterplot of residuals versus estimated height (Figure 5) and confirming the absence of obvious patterns. Consequently, we revised part of the methods (see **page 8, lines 175-177 of Statistical Analysis**), part of the results (see **page 14, line 258 of Results**), and Table 5 (see **page 16**).

We thank the reviewers for their comments and feel that the revised manuscript is much improved. We hope that you find the manuscript suitable for publication in *PLoS One*. We thank you in advance for your kindness and look forward to your favorable reply.

With my best regards,
